# Rifabutin for the Treatment of *Helicobacter pylori* Infection: A Review

**DOI:** 10.3390/pathogens10010015

**Published:** 2020-12-28

**Authors:** Javier P. Gisbert

**Affiliations:** Gastroenterology Unit, Hospital Universitario de La Princesa, Instituto de Investigación Sanitaria Princesa (IIS-IP), Universidad Autónoma de Madrid (UAM), and Centro de Investigación Biomédica en Red de Enfermedades Hepáticas y Digestivas (CIBEREHD), 28006 Madrid, Spain; javier.p.gisbert@gmail.com; Tel.: +34-9130-93911; Fax: +34-9152-04013

**Keywords:** *Helicobacter pylori*, *H. pylori*, rifabutin, treatment, rescue

## Abstract

Nowadays, apart from having to know first-line *Helicobacter pylori* eradication regimens well, we must also be prepared to face treatment failures. The aim of this review is to summarize the role of rifabutin in the management of *H. pylori* infection. Bibliographical searches were performed in PubMed. Data on resistance and efficacy of rifabutin-containing regimens on *H. pylori* eradication were meta-analyzed. Mean *H. pylori* rifabutin resistance rate (39 studies, including 9721 patients) was 0.13%; when studies only including patients naïve to *H. pylori* eradication treatment were considered, this figure was even lower (0.07%). Mean *H. pylori* eradication rate (by intention-to-treat) with rifabutin-containing regimens (3052 patients) was 73%. Respective cure rates for second-, third-, fourth- and fifth-line therapies, were 79%, 69%, 69% and 72%. Most studies administered rifabutin 300 mg/day, which seemed to be more effective than 150 mg/day. The ideal length of treatment remains unclear, but 10–12-day regimens are generally recommended. Adverse events to rifabutin treatment in *H. pylori* studies were relatively infrequent (15%), and severe adverse events were exceptional (myelotoxicity was the most significant, although always reversible). In summary, rifabutin-containing therapy represents an encouraging strategy generally restricted, at present, to patients where previous (usually multiple) eradication regimens have failed.

## 1. Introduction

*Helicobacter pylori* (*H. pylori*) is a worldwide infection that is the main cause not only of gastritis, but also of peptic ulcer disease and gastric cancer [1]. The most commonly used first-line therapies―combining a proton pump inhibitor (PPI) plus two antibiotics, generally including clarithromycin and either amoxicillin or metronidazole―currently fail in more than 20–30% of cases [2]. The major factor affecting our ability to eradicate *H. pylori* infection is antibiotic resistance, mainly to clarithromycin, which is increasing in many geographic areas [3].

At present, a rescue regimen including a quadruple combination of a PPI, bismuth, tetracycline, and metronidazole is generally used as the optimal second-line approach after initial *H. pylori* eradication failure [4,5,6]. However, this treatment fails to eradicate the infection in at least 20% of cases [7,8,9,10]. In addition, this regimen is associated with a high incidence of adverse events, is relatively complex, and many countries are currently experiencing a general unavailability of tetracycline and/or bismuth. On the other hand, quinolone-based rescue regimens, mainly including levofloxacin, achieve a mean eradication rate of only approximately 80%, probably due to the increasing *H. pylori* resistance to quinolones [7,8,9,11,12,13].

Consequently, in some cases, *H. pylori* infection persists even after two or more eradication treatments, and these patients constitute a therapeutic dilemma. Currently, a standard third/fourth-line therapy is lacking, and international guidelines recommend performing culture in these patients to select a rescue treatment according to microbial sensitivity to antibiotics, but this strategy is not practical [14]. Therefore, the evaluation of drugs without cross-resistance to macrolides, nitroimidazole or quinolones as components of retreatment combination therapies seems to be worthwhile [15,16].

Rifabutin is a rifamycin-S derivative, commonly used to treat *Mycobacterium avium* and *Mycobacterium intracellulare*, also referred to as *Mycobacterium avium-intracellulare* complex (MAC) in human immunodeficiency virus (HIV)-infected patients [17,18]. The in vitro sensitivity of *H. pylori* to this antibiotic is high, and it does not share resistance to clarithromycin, metronidazole or levofloxacin [15,16,19,20]. Furthermore, the selection of resistant *H. pylori* strains has been low in experimental conditions. Consequently, rifabutin-based rescue therapies represent a potential and attractive strategy for *H. pylori* eradication failures [21].

The aim of the present review is to summarize the role of rifabutin in the management of *H. pylori* infection. We will review the following topics: (1) rifabutin’s general antimicrobial activity and mechanism of action; (2) pharmacokinetics and pharmacodynamics of rifabutin; (3) prevalence of resistance of *H. pylori* to rifabutin; (4) efficacy of rifabutin for *H. pylori* eradication; (5) optimization strategies aimed to increase the efficacy of rifabutin-based therapies; (6) safety of rifabutin; and, finally, (7) limitations of rifabutin for the treatment of *H. pylori* infection.

## 2. Bibliographic Search and Statistical Analysis

A systematic bibliographic search was performed to identify studies evaluating the role of rifabutin in the management of *H. pylori* infection. An electronic search was conducted in PubMed up to October 2020 using the following algorithm (all fields): rifabutin AND (*Helicobacter pylori* OR *H. pylori*). In addition, the reference lists from the selected articles were reviewed to identify additional studies of potential interest. Articles published in any language were included.

For each study, the variables extracted evaluating the prevalence of rifabutin resistance were as follows: author, year of publication, country, pre- or post-treatment setting, number of patients, and resistance rate. On the other hand, the variables extracted evaluating the efficacy of rifabutin-containing treatments for each study were: author, publication year, country, therapy combination (drugs and doses), treatment duration, number of included patients, number of (previous) failed treatments, type of (previous) failed treatments, eradication rate, and adverse events rate.

The main outcome of interest was the successful eradication of *H. pylori* at least four weeks after completion of treatment, as assessed by at least one reliable diagnostic method (that is, histology, urea breath test or monoclonal stool antigen test); we excluded studies that used only serology testing for the detection of *H. pylori*. Analysis of *H. pylori* eradication efficacy was considered on an intention-to-treat basis (that is, including all eligible patients enrolled in the study regardless of compliance with the study protocol; patients with unevaluable data were assumed to have been unsuccessfully treated). Subanalyses of the data were performed by number of previous failed treatments and by duration of the rifabutin regimen.

Data of variables (including rifabutin resistance, efficacy, and safety of rifabutin-containing regimens on *H. pylori* eradication) were combined using the generic inverse variance method, which involves a weighted average of the effect estimates from the individual studies. The meta-analysis was performed using Review Manager (Cochrane Collaboration). If the results were homogeneous, a fixed effect model was used, and if the results were heterogeneous (*I*^2^ > 50%), a random effect model (DerSimonian and Laird) was applied.

## 3. Rifabutin General Antimicrobial Activity and Mechanism of Action

Rifabutin is structurally related to, and shares many of the properties of, rifampin (rifampicin) [22]. Rifabutin has a broad spectrum of antimicrobial activity, against mycobacteria, a variety of gram-positive and gram-negative bacteria, *Toxoplasma gondii,* and *Chlamydia trachomatis*. It is also active against *Mycobacterium leprae*, *Mycobacterium tuberculosis,* and atypical mycobacteria [22].

Rifabutin is generally more active in vitro than rifampicin against rifampicin-susceptible isolates of *M. tuberculosis*. It is also active against some rifampicin-resistant isolates, although there is substantial cross-resistance in vitro between the two drugs [23].

Rifabutin inhibits the β-subunit of *H. pylori* DNA-dependent RNA polymerase encoded by the *rpoB* gene [24]. Laboratory mutants of *H. pylori*, which are obtained after multiple serial passages in vitro and exhibit amino acid exchanges in codons 524 to 545 or in codon 585 of the *rpoB* gene, are resistant to rifabutin [24,25,26]. As an example, a low rate of resistance (0.24%) to rifabutin was noted in *H. pylori* strains isolated from 414 Japanese patients, and the only rifabutin resistant strain detected showed a point mutation in the *rpoB* gene and was isolated from a patient with a history of rifampin treatment for pulmonary tuberculosis [27].

It has been shown that past rifampicin usage is closely linked to high minimal inhibitory concentrations (MICs) of rifabutin and the prevalence of point mutations in *rpoB* gene, suggesting a cross-resistance between both antibiotics [28]. Therefore, although rifabutin is a potent candidate antibiotic for *H. pylori* eradication as a rescue option, careful consideration must be given to rifampicin treatment history prior to *H. pylori* eradication with rifabutin [25,28].

## 4. Pharmacokinetics and Pharmacodinamics of Rifabutin

Rifabutin is a highly lipid-soluble compound with a pKa of 6.9. It is well absorbed when given orally and exhibits high tissue-to-plasma ratios [22]. High concentrations of the drug are attained in all tissues, particularly those of the liver and lung [22].

Rifabutin is chemically stable at a wide pH range and its antibacterial activity is likely not to be hampered by the acidic environment of the stomach [22,24,29,30]. In one in vivo study performed in rats, the concentrations of rifabutin in gastric juice were 10 to 17 times higher than in blood, indicating extensive secretion into the stomach [31]. 

Mean absolute bioavailability is about 20% after a single dose [23]. When rifabutin is administered with food, its absorption is delayed but not decreased [23]. The apparent volume of distribution is about 8 to 9 L/kg, and the extent of binding to plasma proteins varies between 71% and 94% [23].

Rifabutin is extensively metabolized [23,32]. The mean plasma elimination half-life ranges from 32 to 67 h [23]. Although the pharmacokinetic properties of rifabutin are influenced by hepatic and renal impairment, dosage alteration is probably required only in patients with severe renal or hepatic dysfunction [23]. Rifabutin metabolism in liver microsomes may be inhibited by clarithromycin [33].

## 5. Resistance of *H. pylori* to Rifabutin

Antibiotic resistance to *H. pylori* is the main variable affecting the efficacy of current therapeutic regimens, and it is increasing worldwide [3]. Rifabutin shows good intracellular activity against *H. pylori* [34]. *H. pylori* has proven to be highly susceptible in vitro to rifabutin [23,24,29]. At present, the methodology to identify rifabutin resistance is mainly based on microbiological testing. The in vitro MIC of rifabutin against *H. pylori* [22,29,35] was remarkably lower than that found for amoxicillin, clarithromycin, and metronidazole [22,35].

Rifabutin is generally used to cure or prevent MAC disease in patients with advanced HIV infection, and for this reason, the secondary resistance of *H. pylori* to rifabutin is likely to be absent in the general healthy population [36]. Nevertheless, rifabutin resistance has generally been reported only after the administration of high doses for extended durations, such as those required for the treatment of mycobacterial infections. In this respect, the efficacy of rifabutin-containing regimens to treat MAC infection appear to be uninfluenced by prior prophylactic administration of the drug to patients with acquired immune deficiency syndrome (AIDS) [23].

The majority of rifampicin-resistant strains are isolated from patients after treatment failures, suggesting that previous, unsuccessful attempts of eradication seem to be an important risk factor for the development of rifabutin resistance and/or multiresistance [37,38]. In particular, some reports have documented the (exceptional) possibility of resistance to rifabutin, which was largely explained by previous exposure to this antibiotic [28].

An important concern regarding widespread use of rifabutin for treatment of *H. pylori* infection is the emergence of resistance in *Mycobacterium tuberculosis.* However, no correlation has been reported between short-term use of rifabutin for treatment of non-tuberculosis infections, such as those of *H. pylori*, and emergence of resistance [24,39]. Moreover, prior experience has shown that the risk of development of antimicrobial resistance with *H. pylori* is further reduced when it is used with another antimicrobial agent such as amoxicillin [40].

By analogy with clarithromycin resistance, it has been hypothesized that the presence of multiple strains of *H. pylori*, resistant and/or susceptible to the same antibiotic, in the same patient is possible [41]. Thus, several studies have reported the coexistence of the strains of *H. pylori* that determine the mixed pattern in the same patient [42]. In addition, the presence of *H. pylori* hetero-resistance between two biopsy sites (antrum and fundus) was recently shown in one-third of patients with secondary resistance, suggesting the necessity, in these cases, to perform an antimicrobial susceptibility test from both sites of the stomach [43]. Some authors have assessed the relationship between the presence of mixed infection of *H. pylori* and both antimicrobial susceptibility and virulence markers [44]. When different bacteria were isolated in the host, they showed a resistance to rifabutin (10%) that was higher than that of bacteria in single infection (4%) [44].

A recent systematic review of studies, published from 2006 to 2009, on primary *H. pylori* antibiotic resistance demonstrated that the prevalence rate of rifabutin resistance was very low (1.4%), but only two studies were included in the analysis [45]. The prevalence of primary antibiotic resistance of *H. pylori* in 2008 and 2009 in 18 European countries was assessed in another study, and the rate was 1.1% in adults [3]. More recently, in 2015, Ghotaslou et al. reviewed previous studies (published during the last six years) about the *H. pylori* antibiotic resistance, and the rifabutin resistance rate was relatively high (6.7%) [46]. However, in this review, rifampicin and rifabutin resistance rates were not clearly separated; while both antibiotics are structurally related and share many properties [22], and there is substantial cross-resistance in vitro between the two drugs [23], rifabutin is generally more active in vitro [23,38]. The highest (outlier) result was reported in one study where *H. pylori* eradication treatment was previously prescribed and only 16 patients were included [47].

**Figure 1:**
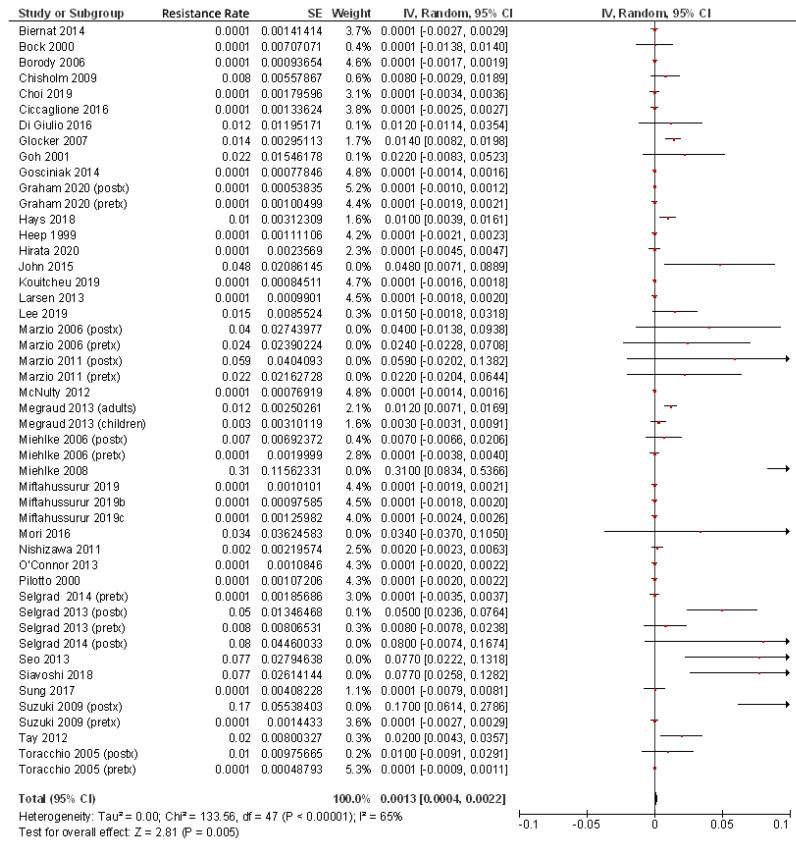
Resistance of *H. pylori* to rifabutin including all patients (both pre- and post-treatment).

**Figure 2:**
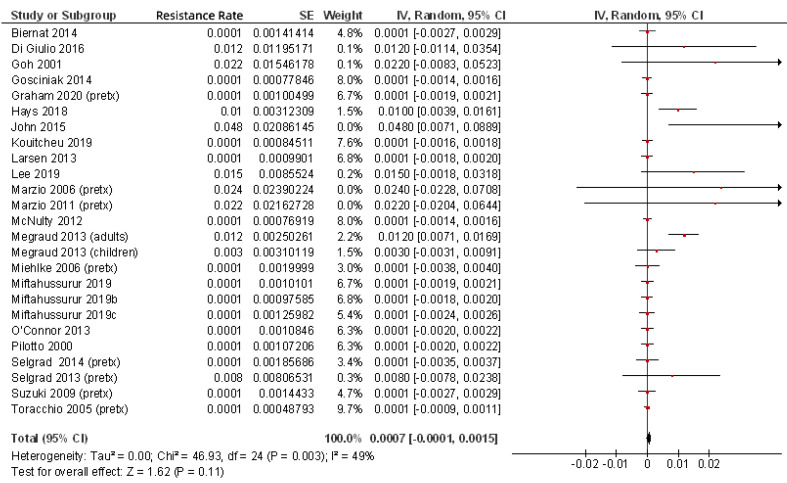
Resistance of *H. pylori* to rifabutin including only patients naïve to *H. pylori* eradication treatment.

Finally, for the present review of the most up to date literature, we summarized in Table 1 all the studies that have evaluated *H. pylori* rifabutin resistance rates [3,24,25,27,28,38,41,47,48,49,50,51,52,53,54,55,56,57,58,59,60,61,62,63,64,65,66,67,68,69,70,71,72,73,74,75,76,77,78]. From these 39 studies, including a total of 9721 patients, an overall rifabutin resistance rate of 0.13% was calculated (Figure 1); however, when only studies including pre-treatment patients (that is, naïve to *H. pylori* eradication treatment) were considered, this figure was even lower (0.07%), as is summarized in Figure 2. Continuous audit of the resistance figures in several populations is needed to keep rifabutin resistance information updated.

## 6. Efficacy of Rifabutin for *H. pylori* Eradication

In 2015, Liu et al. conducted a systematic review and meta-analysis of clinical trials for eradication of *H. pylori* that included a treatment arm with a PPI, rifabutin, and amoxicillin. Twenty-one studies were included, and the overall eradication rate was 70% (by intention-to-treat) [79]. More recently, Gingold-Belfer et al. performed a systematic review of prospective clinical trials (thus excluding retrospective studies) with a treatment arm consisting of PPI, amoxicillin, and rifabutin, and a meta-analysis of randomized controlled trials [80]. The pooled eradication success in the 33 studies selected for subjects treated with rifabutin triple therapy was 71.8%. 

**Figure 3:**
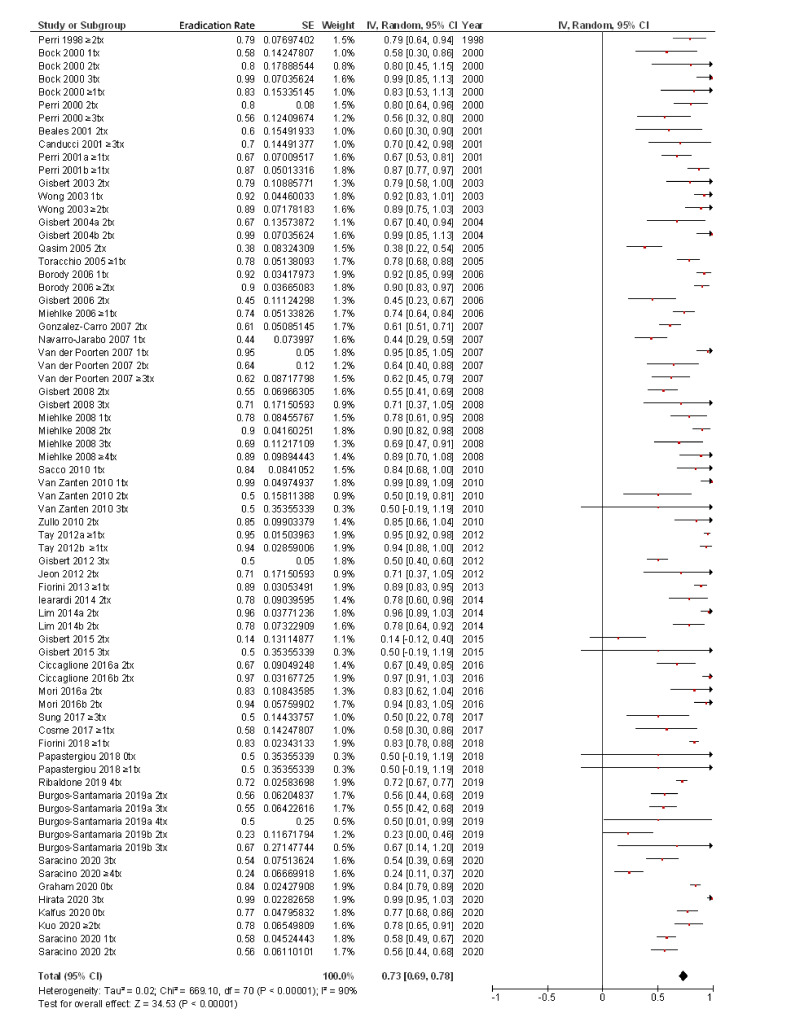
Efficacy (intention-to-treat analysis) of rifabutin-containing therapies for the eradication of *H. pylori.*

Finally, for the present review, to the best of our knowledge the most updated in the literature, all the studies that have evaluated the efficacy of rifabutin-containing regimens for the treatment of *H. pylori* infection are summarized in Table 2 [19,20,36,39,41,47,48,51,52,57,67,68,69,77,78,81,82,83,84,85,86,87,88,89,90,91,92,93,94,95,96,97,98,99,100,101,102,103,104,105,106,107,108]. Overall, from the 3052 patients treated with rifabutin-containing regimens included in Table 2, a mean *H. pylori* eradication rate (by intention-to-treat analysis) of 73% (95% confidence interval (CI), 69–78%) was calculated (Figure 3). In most cases, rifabutin was administered at doses of 300 mg per day (either as 300 mg once a day or as 150 mg twice daily). Duration of treatment was 7 to 14 days in most of the studies. Almost all the protocols included patients with at least one previous *H. pylori* eradication attempt. Type of previous (failed) *H. pylori* eradication treatment varied markedly depending on the study, but in most of the cases included a first-line treatment with a PPI, clarithromycin, and either amoxicillin or nitroimidazole.

Clinical experience with rifabutin for treatment of *H. pylori* infection has focused mainly on patients in whom one or more courses of anti-*H pylori* treatment previously failed [7,8,9,109,110,111]. However, three recent studies evaluated the role of rifabutin in *H. pylori* treatment naïve patients [77,103,107]. The meta-analysis of the efficacy (intention-to-treat) of these studies (including only 307 patients) showed a mean eradication rate of 82% (95% CI, 76–88%). Ultimately, the place of rifabutin as a first-line regimen in anti-*H. pylori* treatment will be determined by factors such as treatment success, adherence, cost, and availability. For the time being, rifabutin may be considered for first-line treatment in regions with high clarithromycin, metronidazole, and levofloxacin resistance (>15%) if bismuth is unavailable [8]. Obviously, head-to-head comparisons against reliable, well-established, and effective first-line regimens will expand efficacy and safety data and further define the role of rifabutin in the treatment of *H. pylori* infections.

**Figure 4:**
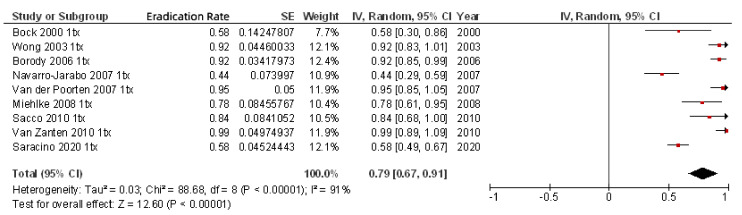
Efficacy (intention-to-treat analysis) of second-line rifabutin-containing therapies for the eradication of *H. pylori* in patients with one previous eradication failure.

**Figure 5:**
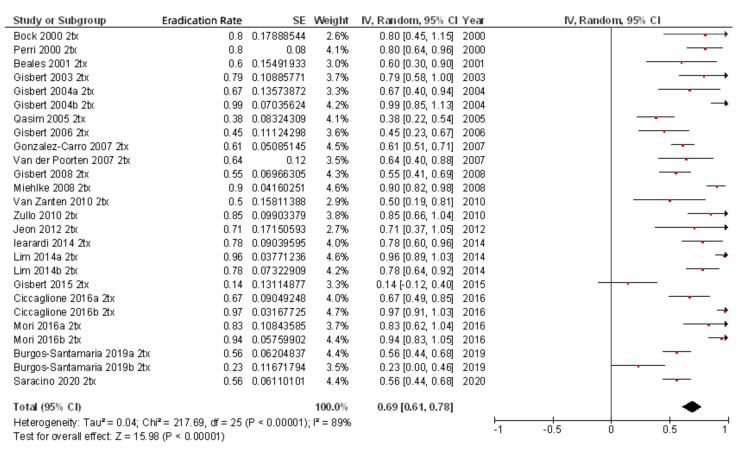
Efficacy (intention-to-treat analysis) of third-line rifabutin-containing therapies for the eradication of *H. pylori* in patients with two previous eradication failures.

As previously noted, most of the studies included rescue regimens after at least one previous eradication failure (Table 2). When only second-line rifabutin-containing therapies administered to the 342 patients with one previous eradication failure were included, the mean eradication rate was 79% (95% CI, 67–91%) (Figure 4). When efficacy of third-line rifabutin-containing therapies for the eradication of *H. pylori* in patients with two previous eradication failures was calculated (including 678 patients), the cure rate was lower (69%; 95% CI, 61–78%) (Figure 5), and similar results were obtained in fourth-line treatment (256 patients; 69% cure rate; 95% CI, 50–88%). Finally, as a fifth-line rescue regimen (including 306 patients), the rifabutin-containing regimen was still quite effective (72%; 95% CI, 67–77%).

**Figure 6:**
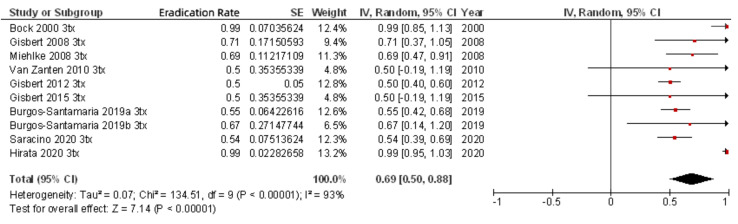
Efficacy (intention-to-treat analysis) of fourth-line rifabutin-containing therapies for the eradication of *H. pylori* in patients with three previous eradication failures.

It has been suggested that rifabutin efficacy decreases with increasing number of previous (failed) therapies, perhaps due to patients who failed eradication therapy and possibly harbored *H. pylori* strains that were more refractory to eradication treatment [39,108]. Accordingly, in the meta-analysis by Gingold-Belfer et al. previously mentioned, treatment success with rifabutin triple therapy was 82% in treatment-naïve patients, 73% in patients receiving second-line treatment, and 64% when this regimen was prescribed as a third-line regimen [80]. However, as summarized in Figure 6, where studies assessing the efficacy of rifabutin in fourth-line regimens are included, quite favorable results may be obtained even after three previous eradication failures. Thus, the mean eradication rate in this scenario was approximately 70%. Accordingly, the efficacy of rifabutin treatment was not significantly influenced by the number of previous treatment failures in a recent study: eradication rates in patients with one, two, three, and four or more previous failures were 78.3%, 89.6%, 68.6%, and 88.9%, respectively (non-statistically significant differences) [47]. 

Just a few studies have directly compared—that is, in the same protocol of *H. pylori* eradication—rifabutin vs. other antibiotics [36,51,86,87,91,104]. Firstly, few studies have compared a PPI, amoxicillin, and rifabutin triple combination with the commonly used bismuth-containing quadruple regimen. Perri et al. [36] were the first to perform a randomized study where patients who failed eradication after standard triple therapy were treated for 10 days with a PPI, amoxicillin, and rifabutin (300 mg/24 h), or with bismuth quadruple therapy. On intention-to-treat analysis, eradication rates were 87% for rifabutin-containing therapy and 67% for bismuth quadruple therapy (these differences being statistically significant). More recent studies have shown better results with the rifabutin-triple [87] or bismuth-quadruple [91,104,108] regimen. Finally, the rifabutin-levofloxacin regimen has also been compared with the bismuth quadruple treatment for second-line treatment, reporting similar high eradication rates (91%) and similar good compliance (>95%) [86].

Miehlke et al., compared the rifabutin regimen with other treatments different to the bismuth-based quadruple regimen [51]. Patients infected with *H. pylori* strains resistant to both clarithromycin and metronidazole were randomized to receive esomeprazole, rifabutin, and amoxicillin for 7 days, or omeprazole 40 mg and amoxicillin 1000 mg, each given three times daily for 14 days. Eradication rates (intention-to-treat) were 74% and 70%, respectively. Therefore, they concluded that high-dose omeprazole/amoxicillin dual regimen and rifabutin triple therapy are comparable for rescue therapy.

Finally, other studies directly compared rifabutin to levofloxacin as third-line therapy for *H. pylori*. In a first study, a statistically significant superiority for the levofloxacin regimen was shown, although it should be noted that the eradication rate of 45% for rifabutin triple therapy in this study was surprisingly low [89]. In another study, on the contrary, higher cure rates were achieved with the rifabutin regimen (in this case, it was the eradication rate of the levofloxacin regimen, only 57%, which was unexpectedly low) [97].

Finally, it should be noted that rifabutin therapy was highly effective even when applied to *H. pylori* infection with primary resistance to clarithromycin or metronidazole (or both) [41,48,49,51,52,77,83,86], and even in patients with triple resistance to these two antibiotics plus quinolones [20], which is the usual scenario after several eradication therapy failures. In this respect, some authors evaluated, in the same study, different regimens after failure of two or more eradication treatments and achieved a final (overall) eradication rate of almost 100% [83,92,106,112,113,114,115,116,117]. This emphasizes the recommendation that in designing a treatment strategy we should prescribe two or more therapies which, if used consecutively, come as close to the 100% cure rate as possible [118]. In this respect, the aforementioned studies underline the fact that a wider perspective of the benefits of retreating *H. pylori* infection can be obtained if cumulative eradication rates, and not only absolute figures, with successive retreatments are taken into account.

## 7. How to Optimize Rifabutin-Based Treatments for *H. pylori*

In this section we will review the optimization strategies aimed to increase the efficacy of rifabutin-based *H. pylori* eradication therapies.

### 7.1. Rifabutin Dose and Frequency

Almost all studies administered rifabutin 300 mg/day for treating *H. pylori* infection. The *H. pylori* cure rate in some (few) studies prescribing rifabutin 150 mg/day was only approximately 40–70% [36,108], although other studies reported higher eradication rates [20,52,99]. A single randomized study directly compared both doses [36]: patients were treated for 10 days with pantoprazole, amoxicillin, and rifabutin 150 mg once daily or 300 mg once daily. Eradication rates were 67% in the rifabutin 150 mg group and significantly higher (87%) in the rifabutin 300 mg group. On the other hand, other authors showed that 12 days of half the dose of rifabutin (150 mg daily) in combination with increasing frequency of dosing with amoxicillin (1 g/8 h) and pantoprazole (80 mg/8 h) achieved an eradication rate as high as 91% [52]. Furthermore, by increasing the dosage of amoxicillin to 1.5 g/8 h for 12 days, an excellent overall eradication rate (97%) was achieved. These data suggest that frequent dosing of a high-dose amoxicillin and a double dose PPI in the presence of low-dose rifabutin (150 mg daily) are critical in increasing the efficacy of triple rifabutin-based therapy [52].

The incidence of adverse events is probably related to the rifabutin dose, with events reported more frequently in patients treated with doses of ≥450 mg daily than in those receiving 300 mg daily, and in those receiving 300 mg compared with 150 mg daily [23].

Finally, regarding the dose of amoxicillin, a meta-analysis by Gingold-Belfer et al. found that treatment was more likely to be successful when daily amoxicillin dose was ≥3000 mg [80].

### 7.2. Duration of Treatment

The ideal length of treatment for the rifabutin regimen remains unclear. In some reports, a 7-day course is equally efficacious compared to 10- to 14-day regimens, while others have found that the 7-day duration dramatically reduced the efficacy, with eradication rates at only 44% [91]. Although some studies have suggested that rifabutin treatment could be more likely to be successful when treatment duration is 14 days [80], many other studies have shown that therapy between 12 and 14 days has yielded results similar to the 10-day course and are likely to increase the incidence of adverse events [39]. Recently, a randomized controlled trial compared 10-day vs. 14-day eradication therapy with esomeprazole 20 mg/6 h, amoxicillin 500 mg/6 h, and rifabutin 300 mg/24 h [67]. Intention-to-treat eradication rates were 83% for the 10-day group and 94% for the 14-day group, respectively. Therapy was stopped due to adverse events in 8% and 29% of patients in the 10-day and 14-day groups, respectively. Therefore, the authors concluded that the 14-day therapy resulted in successful eradication in over 90% of patients, but the 10-day treatment may be enough to obtain a successful eradication rate, considering the tolerability of therapy [67]. Accordingly, from studies included in Table 2 and Figure 3, a mean *H. pylori* eradication rate of 79% for the 14-day regimen was calculated, while the corresponding figure for the 10-day regimen was similar (73%). In summary, current evidence suggests that 10 days may be more effective than 7 days, but no clear additional benefit has been shown with 14 days, which may increase the side effect burden [7,9,111].

### 7.3. Type and Dose of Antisecretory Drug

In a recent study, *H. pylori*-infected patients with two previous eradication failures were randomly assigned to receive either lansoprazole 30 mg/12 h or lansoprazole 60 mg/12 h, together with amoxicillin (1 g/8 h) and rifabutin (150 mg/12 h) for 7 days [98]. Eradication rates were higher in the high-dose PPI group (96% vs. 78%). Therefore, a key factor to successful rescue therapy with rifabutin-amoxicillin-PPI regimen may be to increase doses of PPI. Accordingly, in the meta-analysis by Gingold-Belfer et al., rifabutin treatment was more likely to be successful when daily PPI dose was ≥80 mg [80].

In this same line, studies on rifabutin-based regimens with a potassium-competitive acid blocker such as vonoprazan instead of PPIs may additionally improve the success of the rifamycin-containing eradication therapy. In a recent study, patients who failed *H. pylori* eradication by clarithromycin-based first-line, metronidazole-based second-line, and sitafloxacin-based third-line therapies were prescribed vonoprazan (20 mg), amoxicillin (750 mg), and rifabutin (150 mg) twice daily for 10 days, and *H. pylori* eradication was confirmed in all patients (19/19; 100%) [78].

### 7.4. Addition of Bismuth

Bismuth has an additive effect with antibiotics, overcomes levofloxacin and clarithromycin resistance, and its efficacy is not affected by metronidazole resistance [119,120]. In addition, bismuth is one of the few antimicrobials to which resistance is not developed [121,122]. Bismuth exerts its antibacterial action mainly by preventing bacterial colonization and adherence to gastric epithelium and by binding toxins produced by *H. pylori* [123]. In addition, bismuth decreases mucin viscosity, reduces the bacterial load, and has a synergistic effect with antibiotics [119]. Therefore, combining bismuth and rifabutin in the same regimen is suggested as a promising option.

A combination of a triple therapy with PPI-amoxicillin-rifabutin but adding bismuth and thus converting this triple regimen into a quadruple one, has recently been evaluated, with encouraging results [57,68,100]. Ciccaglione et al. reported that the addition of bismuth to a triple therapy that included PPI, amoxicillin, and rifabutin in patients treated for the third time for *H. pylori* infection, resulted in 30% therapeutic gain compared to rifabutin-based triple therapy alone [68].

Finally, this bismuth-rifabutin regimen has recently been evaluated in the context of the “European Registry on *H. pylori* Management” (Hp-EuReg), an international multicenter prospective non-interventional registry starting in 2013 aimed to evaluate the decisions and outcomes in *H. pylori* management by European gastroenterologists [124]. Thirty European countries, with over 300 recruiters, are actively participating in this project, where patients are managed and registered according to their routine clinical practice [10]. To assess the effectiveness of empirical rescue therapies on third and subsequent lines in Europe, 1782 rescue treatments were evaluated in this registry. The effectiveness of the rifabutin-based quadruple regimen (that is, a PPI, rifabutin, amoxicillin, and bismuth) in these very refractory cases was 59% (by intention-to-treat) [125].

### 7.5. All-in-One Single Capsule Including Omeprazole, Amoxicillin, and Rifabutin

A recent development is the 2019 approval by the United States Food and Drug Administration (FDA) of a combination product (Talicia^®^; RedHill Biopharma, Raleigh, NC, USA) containing omeprazole, rifabutin, and amoxicillin. This is the first and only FDA-approved rifabutin-based *H. pylori* therapy. Potentially, it may improve patient compliance with treatment because of its relative simplicity. In the “ERADICATE Hp2” trial, this combination successfully eradicated *H. pylori* in 84% of patients compared to 58% of those who received the same doses of omeprazole and amoxicillin, but without rifabutin [77]. The recommended dose for this product is four capsules taken three times daily for 14 days; total daily doses are omeprazole 120 mg, rifabutin 150 mg, and amoxicillin 3 g.

A limitation of the aforementioned study is that it was only conducted in the United States and excluded persons of Asian descent. The higher effectiveness of *H. pylori* eradication therapy reported in some studies in Asian populations is possibly due to genetic factors. For example, CYP2C19 polymorphism associated with poor PPI metabolism is more common in Asian populations [80]. This phenotype leads to higher serum PPI levels and more potent gastric acid inhibition and may partially account for the superior eradication rate [126]. While this may lead to a higher bactericidal activity of amoxicillin, CYP2C19 polymorphism is unlikely to affect rifabutin efficacy, which is chemically stable at a wide pH range [80].

## 8. Tolerability of Rifabutin

**Figure 7:**
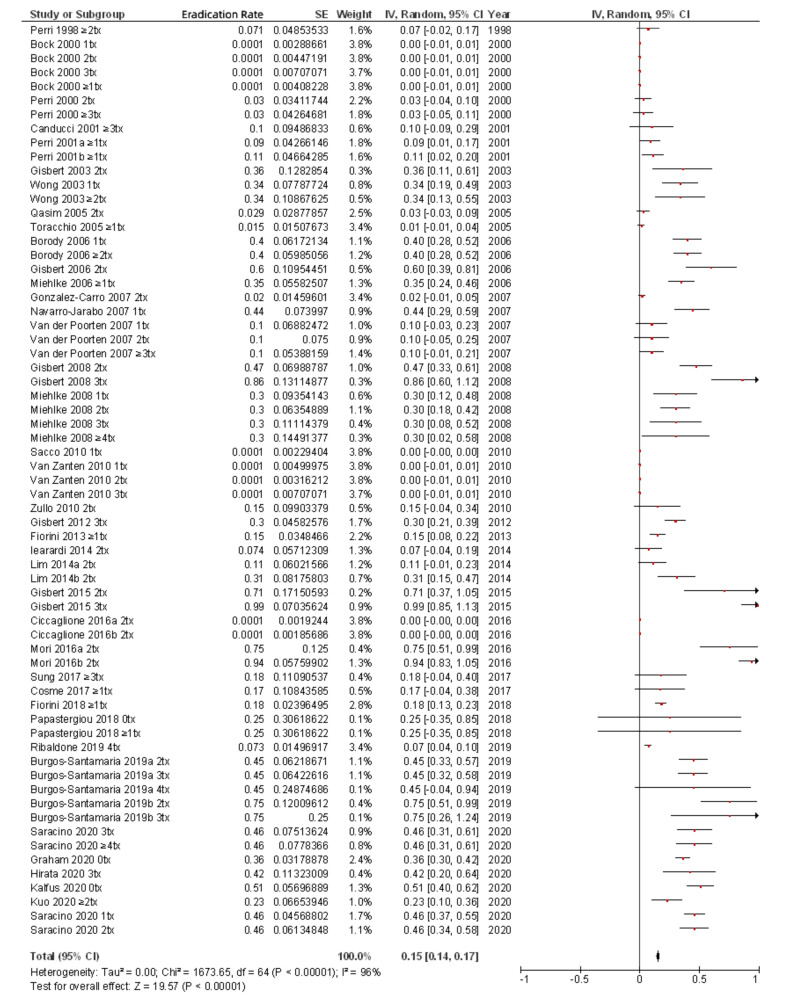
Adverse events of rifabutin-containing therapies.

The overall incidence rate for the occurrence of at least one adverse event (51%) was comparable to placebo (50%) in patients with AIDS treated prophylactically with rifabutin 300 mg daily [23]. Myalgia and taste perversion were, however, significantly more frequent with rifabutin than with placebo [23]. Other reported adverse effects include rash (3%), nausea/vomiting (0.4%), neutropenia (0.4%), anemia (0.4%) and, rarely, impairment of liver function [23,127]. In studies where rifabutin is administered to treat non-*H. pylori* infections, the rate of adverse events is reported as approximately 25–30% [79,80,128]. In our updated meta-analysis of all studies including rifabutin (Table 2), mean rate of adverse effects was 15% (95% CI, 14–17%) (Figure 7), which seems to be a reasonable figure when compared with other well-established eradication regimens, such as the bismuth and non-bismuth quadruple therapies [129]. Furthermore, severe adverse events with rifabutin are exceptional; in a recent review [80], only one severe adverse event was reported (diabetic ketoacidosis) [77].

Myelotoxicity is the most significant adverse event of rifabutin [130,131]. Overall, this complication is rare and is far more likely when high dose (600 mg/day) and prolonged duration therapy is used [130,131]. Although several cases of myelotoxicity have been reported during *H. pylori* therapy [39,41,82,84,86,88,89,96,99], this adverse event was not reported in most studies evaluating rifabutin for *H. pylori* infection. In the studies reporting this complication, myelotoxicity was observed in 1.5% to 3% of patients [39,41,86,88,96], although in some studies the incidence was higher [84,89]. In the meta-analysis by Gingold-Belfer et al., neutropenia was addressed in 27 studies, of which 19 studies reported no cases of neutropenia and eight studies reported at least one case; in total, only 17 patients developed neutropenia across all studies [80].

All patients from the literature recovered from myelotoxicity uneventfully in just a few days, although it took 15 days to normalize white cell count after rifabutin discontinuation in one case [84]. In some cases, the myelotoxicity was clinically apparent with fever [39,84]. However, there are no reports of infection or other adverse outcomes related to reduced white cell count, at least in the scenario of *H. pylori* treatment [39,41,82,84,86,88,89,96].

It has been suggested that during short-term rifabutin treatment blood cell count should be checked at any suspicious symptom. Moreover, some authors have recommended performing systematic blood controls in all patients receiving rifabutin, despite being asymptomatic [85,89]. However, others suggest that a practical approach may be to check the full blood count only if fever or other signs of systemic toxicity occur.

Only a few studies have directly compared—in the same protocol—the safety of rifabutin and other antibiotics in *H. pylori* eradication regimens. Firstly, some studies have compared a triple combination of rifabutin together with a PPI and amoxicillin vs. the widely used bismuth quadruple regimen [36,51,86,91]. As an example, in the study by Perri et al. [36], side effects were less frequent in rifabutin-treated patients than in those on bismuth quadruple therapy. Thus, approximately 15% of patients on quadruple therapy experienced moderate to severe side effects, and 6% had to discontinue treatment. In contrast, in the rifabutin-treated groups, no moderate or severe side effects were observed, and no patients discontinued treatment because of side effects.

Other studies compared rifabutin treatment with other regimens different to the bismuth quadruple therapy. Miehlke et al. [51] compared rifabutin-based triple therapy vs. high-dose dual therapy for rescue treatment of *H. pylori*. Patients were randomized to a PPI, rifabutin, and amoxicillin for 7 days, or to a PPI and amoxicillin 1000 mg/8 h for 14 days. Premature discontinuation of treatment occurred in 2% and 5% of patients, respectively. Finally, one study directly compared rifabutin to levofloxacin as third-line therapy for *H. pylori*, and adverse events were reported in 60% and 50% of cases, respectively [89].

## 9. Limitations of Rifabutin Treatment

Several concerns remain regarding rifabutin for *H. pylori* eradication treatment. First, this drug is quite expensive. Second, as previously reviewed, severe leucopenia has been reported in some (although exceptional) cases. Finally, although an argument raised consistently against the wider use of rifabutin is the concern regarding propagation of resistance, especially in mycobacterial species, it should be taken into account that the major use of rifabutin is for treatment of tuberculosis and other mycobacteria especially in the setting of immunodeficiency or HIV infection [39]. Acquired rifabutin resistance has been noted in these cohorts, but only when associated with CD4 counts <100 cells/mm^3^ and when intermittent dosing is used [132]. Even in the setting of prolonged tuberculosis treatment, continuous daily rifabutin has led to negligible rates of resistance [132,133]. Moreover, no reports have yet to definitively link rifabutin resistance with short course treatment of rifabutin for *H. pylori* or other non-mycobacterial indications [24,39].

## 10. Conclusions

Even with the current most effective treatment regimens, a relevant proportion of patients will fail to eradicate *H. pylori* infection. Nowadays, apart from having to know first-line eradication regimens well, we must also be prepared to face treatment failures. In this context, rifabutin has potential utility against *H. pylori* as this antibiotic shows good in vitro activity against this microorganism, and the prevalence rate of rifabutin resistance is very low (less than 1%).

Mean *H. pylori* eradication rate (overall results, by intention-to-treat analysis) with rifabutin-containing regimens was 73%. Respective cure rates for second-, third-, fourth-, and fifth-line rifabutin therapies, were 79%, 69%, 69%, and 72%, respectively. Almost all studies administered rifabutin 300 mg/day for treating *H. pylori* infection, which seems to be more effective than 150 mg/day. The ideal length of treatment for rifabutin regimen remains unclear, but 10- to 12-day regimens are usually recommended.

The mean rate of adverse effects to rifabutin treatment in *H. pylori* studies was relatively low (15%), and severe adverse events are exceptional. Myelotoxicity is the most significant adverse event of rifabutin during *H. pylori* therapy, although this complication was rare, always reversible, and without clinical consequences (such as infection) in the setting of *H. pylori* treatment.

Regarding the positioning of rifabutin regimens, clinical experience with rifabutin for treatment of *H. pylori* infection has focused mainly on patients in whom one or more courses of anti-*H pylori* treatment have previously failed. Since resistance to rifabutin is practically inexistent, and that rifabutin therapy is highly effective even in patients with primary resistance to clarithromycin, metronidazole, and levofloxacin, rifabutin use in an empirical manner as “rescue” therapy without culture in those patients in whom these antibiotics have failed, may be suggested. Recent studies have evaluated the role of rifabutin in *H. pylori* treatment naïve patients, with encouraging results. Nevertheless, the consideration of rifabutin as a novel first-line treatment option for *H. pylori* infection should be carefully weighed against concerns regarding microbial resistance, treatment cost (rifabutin is quite expensive), and the availability and effectiveness of alternative drugs.

At present, therefore, rifabutin is generally restricted to patients where previous (usually multiple) eradication regimens with key antibiotics such as amoxicillin, clarithromycin, metronidazole, tetracycline, and levofloxacin have failed. Thus, it may be suggested that the position of rifabutin in the algorithm of *H. pylori* treatment may be, usually, at a fourth-line rescue regimen. Nevertheless, rifabutin could be also recommended in an earlier scenario, for example, as second- or third-line treatment, if antibiotic resistance (for example to quinolones) is demonstrated or suspected; or even as a first-line treatment in regions with high clarithromycin, metronidazole, and levofloxacin resistance if bismuth is unavailable.

## Figures and Tables

**Table 1:** Resistance of *H. pylori* to rifabutin.

Author	Year	Country	Pre-Treatment (Naïve) or Post-Treatment Setting	Number of Patients	Resistance Rate (%)
Heep [24]	1999	Germany	Post (mostly)	81	0
Bock [48]	2000	Germany	Post (R)	2	0
Pilotto [49]	2000	Italy	Pre	87	0
Toracchio [41]	2005	Italy	Pre	420	0
Toracchio [41]	2005	Italy	Post (C and M; no R)	104	1
Marzio [50]	2006	Italy	Pre	41	2.4
Marzio [50]	2006	Italy	Post	51	4
Miehlke [51]	2006	Germany	Pre	145	0.7
Miehlke [51]	2006	Germany	Post (C and M; no R)	25	0
Borody [52]	2006	Australia	Post	114	0
Suzuki [28]	2009	Japan	Pre	48	0
Suzuki [28]	2009	Japan	Post	46	17
Chisholm [53]	2007	UK	Post (70%)	255	0.8
Glocker [38]	2007	Germany	Post (R in some)	1585	1.4
Miehlke [47]	2008	Germany	Post (R)	16	31
Goh [54]	2011	Malaysia	Pre	90	2.2
Marzio [55]	2011	Italy	Pre	46	2.2
Marzio [55]	2011	Italy	Post	34	5.9
Nishizawa [27]	2011	Japan	Pre and post	414	0.2
McNulty [56]	2012	UK	Pre and post	169	0
Tay [57]	2012	Australia	Post	306	2
Megraud(adults) [3]	2013	Europe	Pre	1893	1.2
Megraud(children) [3]	2013	Europe	Pre	311	0.3
O’Connor [58]	2013	Ireland	Pre	85	0
Larsen [59]	2013	Norway	Pre	102	0
Selgrad [60]	2013	Germany	Pre	122	0.8
Selgrad [60]	2013	Germany	Post	262	5
Seo [61]	2013	Korea	-	91	7.7
Gosciniak [62]	2014	Poland	Pre	165	0
Biernat [63]	2014	Poland	Pre	50	0
Selgrad [64]	2014	Germany	Pre	29	0
Selgrad [64]	2014	Germany	Post	37	8
John [65]	2015	Qatar	Pre	105	4.8
Di Giulio [66]	2016	Italy	Pre	83	1.2
Mori [67]	2016	Japan	Post	25	3.4
Ciccaglione [68]	2016	Italy	Post	56	0
Sung [69]	2017	Korea	Post	6	0
Hays [25]	2018	France	Pre and post	1015	1
Siavoshi [70]	2018	Iran	-	104	7.7
Choi [71]	2019	Korea	Pre and post	31	0
Lee [72]	2019	Korea	Pre	202	1.5
Kouitcheu M. [73]	2019	Cameroon	Pre	140	0
Miftahussurur [74]	2019	Indonesia	Pre	63	0
Miftahussurur [75]	2019	Indonesia	Pre	105	0
Miftahussurur [76]	2019	Nepal & Bangladesh	Pre	98	0
Graham [77]	2020	USA	Pre	345	0
Hirata [78]	2020	Japan	Post	18	0
Graham [77]	2020	USA	Post	99	0

C: clarithromycin; M: metronidazole; R: rifabutin; Pre: pre-treatment (naïve) setting (that is, before *H. pylori* eradication has been administered); Post: post-treatment setting (that is, when *H. pylori* eradication has been previously prescribed).

**Table 2:** Rifabutin-containing therapies for the eradication of *H. pylori*.

Author	Year	Country	Drugs and Doses	Duration of Treatment (Days)	Number of Patients	Number of Previous Failed Treatments	Type of Previous Treatments	Eradication Rate ¶ (%)	Adverse Events (%)
Perri [81]	1998	Italy	Rifabutin 300 mg/24 h Amoxicillin 1 g/12 h Pantoprazole 40 mg/12 h	7	28	≥2	PPI-containing tx	79	7.1
Bock [48]	2000	Germany	Rifabutin 150 mg/12 h Amoxicillin 1 g/12 h Lansoprazole 30 mg/12 h	7	12	1	PPI+A- and C-containing tx	58	0
Bock [48]	2000	Germany	Rifabutin 150 mg/12 h Amoxicillin 1 g/12 h Lansoprazole 30 mg/12 h	7	5	2	PPI+A- and C-containing tx	80	0
Bock [48]	2000	Germany	Rifabutin 150 mg/12 h Amoxicillin 1 g/12 h Lansoprazole 30 mg/12 h	7	2	3	PPI+A- and C-containing tx	100	0
Bock [48]	2000	Germany	Rifabutin 150 mg/12 h Amoxicillin 1 g/12 h Lansoprazole 30 mg/12 h	7	6	≥1	PPI+A- and C-containing tx	83	0
Perri [82]	2000	Italy	Rifabutin 300 mg/24 h Amoxicillin 1 g/12 h Pantoprazole 40 mg/12 h	7	25	2	(1) PPI+C+A (2) PPI+C+M, PPI+C+T	80	3
Perri [82]	2000	Italy	Rifabutin 300 mg/24 h Amoxicillin 1 g/12 h Pantoprazole 40 mg/12 h	7	16	≥3	(1) PPI+C+A (2) PPI+C+A, PPI+C+M, PPI+C+T 3/(4) PPI+A, PPI+C+M, PPI+A+T, PPI+C+T, RBC+A+T, RBC+C, Q	56	3
Beales [83] ^§^	2001	UK	Rifabutin 300 mg/24 h Amoxicillin 1 g/12 h Omeprazole 20 mg/12 h	14	10	2	C- and M-containing tx	60	-
Canducci [84]	2001	Italy	Rifabutin 300 mg/24 h Amoxicillin 1 g/12 h Omeprazole 20 mg/12 h	10	10	≥3	-	70	10
Perri [36]	2001	Italy	Rifabutin 150 mg/24 h Amoxicillin 1 g/12 h Pantoprazole 40 mg/12 h	10	45	≥1	PPI+C+A, PPI+C+M, RBC+C	67	9
Perri [36]	2001	Italy	Rifabutin 300 mg/24 h Amoxicillin 1 g/12 h Pantoprazole 40 mg/12 h	10	45	≥1	PPI+C+A, PPI+C+M, RBC+C	87	11
Gisbert [85]	2003	Spain	Rifabutin 150 mg/12 h Amoxicillin 1 g/12 h Omeprazole 20 mg/12 h	14	14	2	(1) PPI+C+A (2) Q, RBC+T+M	79	36
Wong [86]	2003	China	Rifabutin 300 mg/24 h Levofloxacin 500 mg/24 h Rabeprazole 20 mg/12 h	7	37	1	PPI+C+A, PPI+C+M, PPI+A+M	92	34
Wong [86]	2003	China	Rifabutin 300 mg/24 h Levofloxacin 500 mg/24 h Rabeprazole 20 mg/12 h	7	19	≥2	C-containing tx (100%), M-containing tx (76%)	89	34
Gisbert [87]	2004	Spain	Rifabutin 150 mg/12 h Amoxicillin 1 g/12 h Omeprazole 20 mg/12 h	14	12	2	(1) PPI+C+A (2) RBC+T+M	67	-
Gisbert [87]	2004	Spain	Rifabutin 150 mg/12 h Amoxicillin 1 g/12 h Omeprazole 20 mg/12 h	14	2	2	(1) RBC+C+A (2) RBC+T+M	100	-
Qasim [88]	2005	Ireland	Rifabutin 300 mg/24 h Amoxicillin 1 g/12 h PPI/12 h	10	34	2	(1) PPI+C+A, PPI+C+M (2) PPI+C+A, PPI+C+M, Q	38	2.9
Toracchio [41] ^¥^	2005	Italy	Rifabutin 150 mg/12 h Amoxicillin 1 g/12 h Pantoprazole 40 mg/12 h	10	65	≥1	-	78	1.5
Borody [52]	2006	Australia	Rifabutin 150 mg/24 h Amoxicillin 1–1.5 g/8 h Pantoprazole 80 mg/8 h	12	63	1	C-containing tx	92	40
Borody [52]	2006	Australia	Rifabutin 150 mg/24 h Amoxicillin 1–1.5 g/8 h Pantoprazole 80 mg/8 h	12	67	≥2	C-containing tx	90	40
Gisbert [89]	2006	Spain	Rifabutin 150 mg/12 h Amoxicillin 1 g/12 h Omeprazole 20 mg/12 h	10	20	2	(1) PPI+C+A (2) Q, RBC+T+M	45	60
Miehlke [51] ^§^	2006	Germany	Rifabutin 150 mg/12 h Amoxicillin 1 g/12 h Esomeprazole 20 mg/12 h	7	73	≥1 (24% 1 tx, 52% 2 tx, 24% 3 tx)	PPI+C+A, PPI+C+M, PPI+A+M, Q, PPI+A+L, others	74	35
Gonzalez Carro [90]	2007	Spain	Rifabutin 150 mg/12 h Amoxicillin 1 g/12 h Pantoprazole 40 mg/12 h	10	92	2	(1) PPI+C+A (2) Q	61	2
Navarro-Jarabo [91]	2007	Spain	Rifabutin 150 mg/12 h Amoxicillin 1 g/12 h Omeprazole 20 mg/12 h	7	45	1	PPI+C+A, PPI+C+M	44	44
Van der Poorten [39]	2007	Australia	Rifabutin 150 mg/12 h Amoxicillin 1 g/12 h PPI/12 h	10	19	1	C-containing tx	95	10
Van der Poorten [39]	2007	Australia	Rifabutin 150 mg/12 h Amoxicillin 1 g/12 h PPI/12 h	10	16	2	(1) C-containing tx (2) Q (42% of the cases)	64	10
Van der Poorten [39]	2007	Australia	Rifabutin 150 mg/12 h Amoxicillin 1 g/12 h PPI/12 h	10	31	≥3	(1) C-containing tx (2) Q (42% of the cases) (3) Other	62	10
Gisbert [92]	2008	Spain	Rifabutin 150 mg/12 h Amoxicillin 1 g/12 h Omeprazole 20 mg/12 h	10-14	51	2	(1) PPI+C+A (2) Q, RBC+T+M, PPI+A+L	55	47
Gisbert [92]	2008	Spain	Rifabutin 150 mg/12 h Amoxicillin 1 g/12 h Omeprazole 20 mg/12 h	10-14	7	3	(1) PPI+C+A (2) Q, RBC+T+M (3) PPI+A+L	71	86
Miehlke [47] ^¥^	2008	Germany	Rifabutin 300 mg/24 h Moxifloxacin 400 mg/24 h Esomeprazole 40 mg/24 h	7	24	1	PPI+A, PPI+C, PPI+C+A, PPI+C+M, Q, PPI+A+L	78	30
Miehlke [47] ^¥^	2008	Germany	Rifabutin 300 mg/24 h Moxifloxacin 400 mg/24 h Esomeprazole 40 mg/24 h	7	52	2	PPI+A, PPI+C, PPI+C+A, PPI+C+M, Q, PPI+A+L	90	30
Miehlke [47] ^¥^	2008	Germany	Rifabutin 300 mg/24 h Moxifloxacin 400 mg/24 h Esomeprazole 40 mg/24 h	7	17	3	PPI+A, PPI+C, PPI+C+A, PPI+C+M, Q, PPI+A+L	69	30
Miehlke [47] ^¥^	2008	Germany	Rifabutin 300 mg/24 h Moxifloxacin 400 mg/24 h Esomeprazole 40 mg/24 h	7	10	≥4	PPI+A, PPI+C, PPI+C+A, PPI+C+M, Q, PPI+A+L	89	30
Sacco [93]	2009	Italy	Rifabutin 150 mg/12 h Amoxicillin 1 g/12 h Esomeprazole 20 mg/12 h	10	19	1	PPI+A+Mx	84	0
Van Zanten [94]	2010	Canada	Rifabutin 300 mg/24 h Amoxicillin 1 g/12 h PPI/12 h	7	4	1	-	100	0
Van Zanten [94]	2010	Canada	Rifabutin 300 mg/24 h Amoxicillin 1 g/12 h PPI/12 h	7	10	2	(1) PPI+C+A, PPI+C+M, RBC+C (2) PPI+C+A, PPI+C+M, Q	50	0
Van Zanten [94]	2010	Canada	Rifabutin 300 mg/24 h Amoxicillin 1 g/12 h PPI/12 h	7	2	3	(1) PPI+C+A, Q (2) PPI+A, PPI+C+M (3) PPI+C+A	50	0
Zullo [95]	2010	Italy	Rifabutin 150 mg/12 h Amoxicillin 1 g/12 h Omeprazole 20 mg/12 h	10	13	2	(1) PPI+C+A, PPI+C+M, PPI+A+M (2) PPI+A+L	85	15
Tay [57]	2012	Australia	Rifabutin 150 mg/12 h Amoxicillin 1 g/8 h Ciprofloxacin 500 mg/12 h Rabeprazole 20 mg/8 h	5-7	210	≥1	(1) PPI+C+A +/- others (mostly PPI+C+M)	95	-
Tay [57]	2012	Australia	Rifabutin 150 mg/12 h Bismuth 240 mg/6 h Ciprofloxacin 500 mg/12 h Rabeprazole 20 mg/8 h	7	69	≥1	PPI+C+A +/- others (mostly PPI+C+M)	94	-
Gisbert [96]	2012	Spain	Rifabutin 150 mg/12 h Amoxicillin 1 g/12 h PPI/12 h	10	100	3	(1) PPI+C+A (2) Q (3) PPI+A+L	50	30
Jeong [97]	2012	Korea	Rifabutin 150 mg/12 h Amoxicillin 1 g/12 h PPI/12 h	10	7	2	(1) PPI+C+A (2) Q	71	-
Fiorini [99]	2013	Italy	Rifabutin 150 mg/24 h Amoxicillin 1 g/8 h Esomeprazole 40 mg/12 h	12	105	≥1	-	89	15
Ierardi [100]	2014	Italy	Rifabutin 150 mg/12 h Minocycline 100 mg/12 h Bismuth 120 mg/6 h Rabeprazole 20 mg/12 h	10	21	2	(1) PPI+C+A, sequential (2) PPI+A+L	78	7.4
Lim [98]	2014	Korea	Rifabutin 150 mg/12 h Amoxicillin 1 g/8 h Lansoprazole 60 mg/12 h	7	27	2	(1) PPI+C+A (2) Q	96	11
Lim [98]	2014	Korea	Rifabutin 150 mg/12 h Amoxicillin 1 g/8 h Lansoprazole 30 mg/12 h	7	32	2	(1) PPI+C+A (2) Q	78	31
Gisbert [101] ^#^	2015	Spain	Rifabutin 150 mg/12 h Clarithromycin 500 mg/12 h Omeprazole 20 mg/12 h	10	7	2	(1) PPI+C+M (2) Q	14	71
Gisbert [101] ^#^	2015	Spain	Rifabutin 150 mg/12 h Clarithromycin 500 mg/12 h Omeprazole 20 mg/12 h	10	2	3	(1) PPI+C+M (2) Q (3) PPI+C+L	50	100
Ciccaglione [68]	2016	Italy	Rifabutin 150 mg/12 h Amoxicillin 1 g/12 h Pantoprazole 20 mg/12 h	10	27	2	(1) PPI+C+A (2) PPI+A+M/L/Mx	67	0
Ciccaglione [68]	2016	Italy	Rifabutin 150 mg/12 h Amoxicillin 1 g/12 h Pantoprazole 20 mg/12 h Bismuth 240 mg/12 h	10	29	2	(1) PPI+C+A (2) PPI+A+M/L/Mx	97	0
Mori [67]	2016	Japan	Rifabutin 300 mg/24 h Amoxicillin 500 mg/8 h Esomeprazole 20 mg/8 h	10	12	2	(1) PPI+C+A (2) PPI+A+M	83	75
Mori [67]	2016	Japan	Rifabutin 300 mg/24 h Amoxicillin 500 mg/8 h Esomeprazole 20 mg/8 h	10	17	2	(1) PPI+C+A (2) PPI+A+M	94	94
Sung [69]	2017	Korea	Rifabutin 150 mg/12 h Amoxicillin 1 g/12 h PPI 20 mg/12 h	7-14	12	≥3	-	50	18
Cosme [102]	2017	Spain	Rifabutin 150 mg/12 h Amoxicillin 1 g/12 h Omeprazole 20 mg/12 h	10	12	≥1	-	58	17
Fiorini [20]	2018	Italy	Rifabutin 150 mg/24 h Amoxicillin 1 g/12 h Esomeprazole 40 mg/12 h	12	257	≥1	-	83	18
Papastergiou [103]	2018	Greece	Rifabutin 150 mg/12 h Amoxicillin 1 g/8 h Esomeprazole 40 mg/12 h	7	2	0 (naïve)	-	50	25
Papastergiou [103]	2018	Greece	Rifabutin 150 mg/12 h Amoxicillin 1 g/8 h Esomeprazole 40 mg/12 h	7	2	≥1	-	50	25
Ribaldone [105]	2019	Italy	Rifabutin 150 mg/12 h Amoxicillin 1 g/12 h PPI/12 h	14	302	4	(1) PPI+C+A (2) PPI+C+A+M (3) Q (4) PPI+A+L	72	7.3
Burgos-Santamaría [106]	2019	Spain	Rifabutin 150 mg/12 h Amoxicillin 1 g/12 h Omeprazole 20 mg/12 h	10-14	64	2	(1) PPI+C+A (2) Q	56	45
Burgos-Santamaría [106]	2019	Spain	Rifabutin 150 mg/12 h Amoxicillin 1 g/12 h Omeprazole 20 mg/12 h	10-14	60	3	(1) PPI+C+A (2) Q (3) PPI+A+L (or vice versa)	55	45
Burgos-Santamaría [106]	2019	Spain	Rifabutin 150 mg/12 h Amoxicillin 1 g/12 h Omeprazole 20 mg/12 h	10-14	4	4	(1) PPI+C+A (2) Q (3) PPI+A+L (or vice versa) (4) Other	50	45
Burgos-Santamaría [106] ^#^	2019	Spain	Rifabutin 150 mg/12 h Clarithromycin 500 mg/12 h Omeprazole 20 mg/12 h	10-14	13	2	(1) PPI+C+M (2) Q (or vice versa)	23	75
Burgos-Santamaría [106] ^#^	2019	Spain	Rifabutin 150 mg/12 h Clarithromycin 500 mg/12 h Omeprazole 20 mg/12 h	10-14	3	3	(1) PPI+C+M (2) Q (or vice versa) (3) PPI+C+L	67	75
Saracino [108]	2020	Italy	Rifabutin 150 mg/24 h Amoxicillin 1 g/12 h Esomeprazole 40 mg/12 h	12	119	1	-	58	46
Saracino [108]	2020	Italy	Rifabutin 150 mg/24 h Amoxicillin 1 g/12 h Esomeprazole 40 mg/12 h	12	66	2	-	56	46
Saracino [108]	2020	Italy	Rifabutin 150 mg/24 h Amoxicillin 1 g/12 h Esomeprazole 40 mg/12 h	12	44	3	-	54	46
Saracino [108]	2020	Italy	Rifabutin 150 mg/24 h Amoxicillin 1 g/12 h Esomeprazole 40 mg/12 h	12	41	≥4	-	24	46
Graham [77]	2020	USA	Rifabutin 50 mg/8 h Amoxicillin 1 g/8 h Omeprazole 40 mg/12 h	14	228	0 (naïve)	-	84	36
Hirata [78]	2020	Japan	Rifabutin 150 mg/12 h Amoxicillin 750 mg/12 h Vonoprazan 20 mg/12 h	10	19	3	(1) C-containing tx (2) M-containing tx (3) S-containing tx	100	42
Kalfus [107]	2020	USA	Rifabutin 150 mg/8 h Amoxicillin 1 g/8 h Omeprazole 40 mg/8 h (Talicia^®^ single capsule)	14	77	0 (naïve)	-	77	51
Kuo [19]	2020	China	Rifabutin 150 mg/12 h Amoxicillin 1 g/12 h Esomeprazole 40 mg/12 h	10	40	≥2	-	78	23

PPI: proton pump inhibitor (standard dose); Type of previous treatments: (1) indicates 1st line treatment, (2) indicates 2nd line treatment, and (3) indicates 3rd line treatment; Tx: treatments; C: clarithromycin; A: amoxicillin; Q: bismuth quadruple therapy (PPI, bismuth, tetracycline, and nitroimidazole); RBC: ranitidine bismuth citrate; T: tetracycline; M: metronidazole; L: levofloxacin; Mx: moxifloxacin; S: sitafloxacin. ^¶^ Intention-to-treat analysis. ^§^ Patients infected with *H. pylori* resistant to both clarithromycin and metronidazole/tinidazole. ^¥^ Patients infected with *H. pylori* resistant to both clarithromycin and metronidazole/tinidazole, and susceptible to rifabutin. ^#^ Patients allergic to penicillin.

## Abbreviations

Helicobacter pylori	*H. pylori*
proton pump inhibitor	PPI
